# Soluble Programmed Death Receptor Ligands sPD-L1 and sPD-L2 as Liquid Biopsy Markers for Prognosis and Platinum Response in Epithelial Ovarian Cancer

**DOI:** 10.3389/fonc.2019.01015

**Published:** 2019-10-15

**Authors:** Paul Buderath, Esther Schwich, Christina Jensen, Peter A. Horn, Rainer Kimmig, Sabine Kasimir-Bauer, Vera Rebmann

**Affiliations:** ^1^Department of Gynecology and Obstetrics, University Hospital Essen, Essen, Germany; ^2^Institute for Transfusion Medicine, University Hospital Essen, Essen, Germany

**Keywords:** epithelial ovarian cancer (EOC), soluble PD-L1 (sPD-L1), soluble PD-L2 (sPD-L2), liquid biopsy, biomarkers, platinum therapy, residual tumor burden, circulating tumor cells

## Abstract

**Introduction:** Response to platinum-based therapy is a major prognostic factor in epithelial ovarian cancer (EOC) and reliable prognostic biomarkers are urgently needed to identify patients at high risk. Since ligands of the Programmed Death Receptor-1 (PD-L1 and PD-L2) play a crucial role within the tumor microenvironment for tumorigenesis, we investigated levels of sPD-L1 and sPD-L2 in liquid biopsies of serum samples, and correlated the results with the clinical status, presence of circulating tumor cells (CTCs) and disease outcome in primary EOC patients.

**Methods:** sPD-L1 and sPD-L2 were determined by ELISA in patients (*N* = 83) and healthy females (*N* = 29). Gene expression analysis of EpCAM, MUC-1, CA-125, and ERCC1 was performed by RT-PCR after CTCs enrichment.

**Results:** sPD-L1 was significantly (*p* = 0.0001) increased and sPD-L2 decreased (*p* = 0.003) in EOC patients compared to controls. While enhanced sPD-L1 was associated with residual tumor burden (*p* = 0.022), reduced sPD-L2 levels were related to platinum-resistance (*p* < 0.01) and the presence of ERCC1+ CTCs (*p* < 0.0001). High sPD-L1 levels were associated with a reduced 5 year overall survival (OS, *p* = 0.003) and progression-free survival (PFS, *p* = 0.019). Strikingly, sPD-L1 levels >6.4 pg/ml were indicative of a reduced OS (*p* = 0.035) and PFS (*p* = 0.083) in platinum-sensitive patients, while OS and PFS in platinum-resistant patients did not differ when patients were stratified to this cut-off.

**Conclusions:** Our study highlights sPD-L1 and sPD-L2 as complementary biomarkers reflecting clinical status, treatment response and disease outcome of EOC patients. Especially, sPD-L1 may facilitate the identification of high-risk patients with unfavorable disease outcomes despite platinum-sensitivity arguing for additional therapeutic approaches. As sPD-L1 and sPD-L2 are easily accessible via liquid biopsy, the inclusion of sPD-L1 and sPD-L2 in addition to CTC investigation as markers for risk assessment during patient therapy planning and follow-up appears to be a valuable approach.

## Introduction

Epithelial ovarian cancer (EOC) is the most lethal of gynecological malignancies worldwide (1). In Germany, EOC is responsible for 3.2% of all malignant neoplasms and 5.3% of all cancer deaths (2). Due to the lack of early symptoms, most patients are diagnosed at an advanced stage. Standard therapy consists of radical cytoreductive surgery and a platinum-based chemotherapy. In recent years, the anti-VEGF antibody Bevacizumab has become part of the standard adjuvant treatment for advanced EOC. Despite a high initial response rate, the majority of patients eventually relapse (3), leading to a relative 5-year survival of 41% (2). The platinum-free interval to relapse has been identified as a predictive factor for the response to subsequent platinum-based therapy (4, 5). Patients relapsing within 6 months after initial platinum-based chemotherapy are generally considered platinum-resistant with subsequent platinum-based therapy not being an option. Thus, treatment options for this group of patients are limited, making these women the most challenging to treat. As there is currently no clinically established method of predicting response to first-line platinum-based treatment, reliable predictive biomarkers are urgently needed to estimate the risk of relapse and to improve treatment management. In this regard, it has already been reported that the characterization of disseminated tumor cells (DTCs) in the bone marrow (BM), and circulating tumor cells (CTCs) in blood has identified stem cell-like DTCs and CTCs, tumor cells in epithelial-mesenchymal transition (EMT) as well as resistant cells, and all associated with poor outcomes and clinical platinum resistance [reviewed in Giannopoulou et al. (6)]. Interestingly, we were able to show that immunotherapy based on the intraperitoneal trifunctional bispecific antibody Catumaxomab was successful in the elimination of DTCs and CTCs in patients with advanced EOC (7). Thus, the tumor microenvironment might play a crucial role in tumor control and tumorigenesis of these patients.

The immune-regulatory protein PD-1, expressed by different immune cells, and its ligands PD-L1 and PD-L2, expressed by tumor cells and a variety of immune cells, have gained attention for treatment options in EOC (8). Hitherto, anti-PD-1 and anti-PD-L1 antibodies have already been successfully used in early clinical studies (9). PD-L1 was shown to be primarily expressed by tumor cells and correlated with worst prognosis (10). However, subsequent studies showed contradictory results with PD-L1 being expressed primarily by macrophages resulting in a longer OS (11). Recently, the soluble forms of PD-1 and PD-L1 (sPD-1 and sPD-L1) in serum samples were considered to be effective for the prediction of prognosis and treatment response (12, 13). sPD-L1 plasma levels were shown to be significantly increased in EOC patients compared to women with benign tumors and healthy controls (14). However, studies elucidating the role of sPD-L1 and sPD-L2 in EOC are rare.

Although the cellular expression of the immune checkpoint molecule PD-1 and its ligands PD-L1 and PD-L2 in the tumor microenvironment play crucial roles in tumor control and tumorigenesis, the routine clinical analysis is problematic due to the need to obtain representative biopsies of the entire tumor. Thus, we hypothesized that assessing the soluble forms of PD-L1 and PD-L2 in peripheral blood of EOC patients might represent a feasible approach in the search for “liquid biomarkers” to target patients accordingly.

To introduce sPD-L1 and sPD-L2 as biomarkers for disease status and outcome, we evaluated levels of sPD-L1 and sPD-L2 in sera of 83 primary EOC patients retrospectively and related these results with the presence of CTCs, clinical characteristics including FIGO-stage, tumor grade, lymph node infiltration, presence of metastases, residual tumor burden and platinum resistance as well as PFS and OS.

## Materials and Methods

### Patient Characteristics

A total number of 83 patients diagnosed with histologically confirmed EOC between 2007 and 2014 at the Department of Gynecology and Obstetrics, University Hospital Essen, were analyzed. Histological subtype was serous except for 3 cases with poorly differentiated tumors which could not be assigned to a specific subtype by the pathologist. Written informed consent was obtained from all participants and the study was approved by the local ethics committee (Essen 05-2870 and 17-7859) and performed according to the Declaration of Helsinki. Tumors were classified according to the WHO classification of tumors of the female genital tract. Grading was conducted using the grading system proposed by Silverberg and tumor staging was classified according to the Fédération Internationale de Gynécology et d'Obstétrique (FIGO). The whole study population underwent primary radical surgery. Total abdominal hysterectomy, bilateral salpingo-oophorectomy, infragastric omentectomy, and peritoneal stripping were performed. The most important aim of surgery was to achieve macroscopic complete tumor resection. Radical pelvic and para-aortic lymphadenectomy were only performed if macroscopic complete tumor resection was achieved intraperitoneally following guideline recommendation during the reported time period. All patients received at least six cycles of carboplatinum AUC 5 and paclitaxel 175 mg/m^2^. Tumors were clinically defined as platinum-resistant if they recurred within 6 months after the completion of platinum-based chemotherapy. Any macroscopic residual disease at the end of primary surgery was defined as residual tumor. Inclusion criteria were: histologically confirmed EOC, primary radical surgery, platinum-based chemotherapy, availability of serum-samples, and follow-up information. All patients from the selected time period who met these criteria were included. Chemotherapy was administered postoperatively in all patients during this period. Clinical characteristics of the patients and association to sPD-L1 and sPD-L2 levels are documented in Table 1.

**Table 1:** Patients' characteristics and association to sPD-L1 and sPD-L2 serum-levels.

		***n***	**sPD-L1^†^**	***p***	**sPD-L2^†^**	***p***
FIGO stage	FIGO II	6	3.8; 0.0–9.2	n.s.	1,870; 906–5,925	n.s.
	FIGO III	55	5.7; 0.0–32.9		1,968; 260–6,300	
	FIGO IV	22	8.4; 1.2–24.0		1,773; 712–6,300	
Grading	G1-G2	33	6.8; 0.0–32.9	n.s.	1,710; 675–5,925	n.s.
	G3	50	6.0; 0.0–24.0		1,906; 260–6,300	
Histo-pathological type	Serous	80	6.0; 0.0–32.9	n.s.	1,906; 260–6,300	n.s.
	Non-specified	3	10.6; 9.4–10.6		1,250; 778–1,435	
Progression	No	28	4.3. 0.0–19.7	0.03	1,906; 850–5,925	n.s.
	Yes	55	7.0; 0.0–32.9		1,831; 260–6,300	
Survival	No	40	7.3; 1.1–32.9	0.003	1,712: 260–6,300	n.s.
	Yes	43	4.2; 0.0–23.6		1,919; 850–5,925	
Platinum resistance	No	55	5.9; 0.0–32.9	n.s.	1,919; 686–6,300	0.0096
	Yes	13	4.5; 0.0–13.1		1,338; 352–3,061	
	Unknown	15	7.4; 1.2–21.9		1,919; 260–6,300	
Residual tumor	No	40	5.5; 0.0–21.9	0.022	1,907; 352–6,300	n.s.
	Yes	43	7.5; 0.0–32.9		1,831; 260–6,300	
CTC before therapy	No	60	5.9; 0.0–32.9	n.s.	2,100; 686–6,300	<0.0001
	Yes	22	7.2; 0.0–23.2		1,324; 260–3,019	
	Unknown	1	10.5; 10.5–10.5		778; 778–788	
CTC after therapy	No	21	3.3; 0.0–23.9	n.s.	2,354; 850–6,300	n.s.
	Yes	10	3.7; 0.0–11.9		1,656; 675–3,019	
	Unknown	52	7.4; 0.0–32.9		1,853; 260–6,300	

† *Given as median; minimum—maximum in pg/ml; n.s., not significant; platinum resistance was defined as recurrence <6 months after completion of adjuvant platinum therapy; residual tumor was defined as any macroscopic disease at the end of primary surgery*.

Patients had a median age of 68 (range: 32–98) years, whereas the control cohort consisted of 29 healthy women with a median age of 55 ranging from 35 to 70 years. Overall survival (OS) and progression-free survival (PFS) ranged from 1 to 118 months with a median of 30 months and 1 to 111 with a median of 18 months, respectively.

### Sampling of Serum

Serum samples of healthy women or of EOC patients at the time of first diagnosis were collected and centrifuged for 10 min at 2,500×g. Subsequently, the upper phase was stored at −20°C until usage.

### Assessment of Soluble PD-L1 and PD-L2

Serum concentrations of sPD-L1 and sPD-L2 were measured by commercial ELISA kits (R&D Systems, GmbH, Wiesbaden-Nordenstadt, Germany) using the manufacturer's protocols with minor modifications. In brief, microtiter plates with high binding surface (Costar Corning, Bodenheim, Germany) were coated with anti-human PD-L1 or PD-L2 antibody at 4°C overnight in a final concentration of 4 and 2 μg/ml, respectively. For the detection of bound sPD-L1 or sPD-L2, biotin-coupled polyclonal goat anti-human PD-L1 and PD-2, respectively, was used diluted to 50 ng/ml or 1 μg/ml in phosphate-buffered saline (PBS) supplemented with 1% bovine serum albumin (BSA, AppliChem GmbH, Darmstadt, Germany). Bound detection antibodies were recognized by streptavidin conjugated with horseradish peroxidase being diluted 1:200 in PBS containing 1% BSA; 3,3,5,5-tetramethybenzidine substrate reagent set (Becton Dickinson, Franklin Lakes, USA) was used for visualizing immune complexes. Substrate reaction was terminated using 2N H2SO4 and optical density was measured at 450 nm (Biotek Instruments, Winooski, VT).

All serum samples were tested undiluted. Recombinant PD-L1 and PD-L2 protein fused with Fc portion of human IgG were used as standard reagents. PD-L1 or PD-L2 standard was serially diluted from 0 to 1,250 pg/ml or 0 to 6,000 pg/ml. Quantification of sPD-L1 and sPD-L2 serum levels were performed by four-parameter curve fitting. For sPD-L1 and sPD-L2, the intra-assay coefficients of variations were 6.6 and 5.2%, respectively, whereas the inter-assay coefficients of variations were 15.0% for sPD-L1 and 9.1% for sPD-L2.

### Selection and Detection of CTCs

Ethylenediaminetetraacetic acid blood samples were collected and processed within 4 h for the enrichment of CTCs. In briefly, CTCs were immunomagnetically selected using the AdnaTest *OvarianCancerSelect* (QIAGEN, Hilden, Germany). After RNA isolation, gene expression analysis was done by reverse-transcription (RT) and multiplex RT-PCR, detecting EpCAM, MUC-1, and CA-125 (AdnaTest *OvarianCancerDetect*). ERCC1-transcripts were studied by a singleplex RT-PCR (*n* = 57/83 patients). β-actin served as an internal control. Assays have been described in detail elsewhere (15, 16).

### Statistical Analysis

All statistical analyses were performed using IBM SPSS Statistics Version 24. Continuous and categorical variables were compared using the Mann-Whitney *U*, Kruskal–Wallis test, or Fisher's exact test, as appropriate. Receiver operating curve (ROC) analysis was performed to obtain cut-off values for categorization of continuous patient characteristics into dichotomous variables representing the optimal separation of survival curve by using the BIAS 11.01 software program (http://www.bias-online.de/). Probabilities of OS and PFS were analyzed using the Kaplan-Meier method in combination with the log-rank test implemented in the R package survminer (version 0.4.0; https://CRAN.R-project.or/package=survminer). Starting points were time point of diagnosis (blood collection) and endpoints were death from EOC, progress or relapse of EOC disease (therapy requirement). Differences with a *p*-value < 0.05 were considered statistically significant.

## Results

### Increased sPD-L1 but Decreased sPD-L2 Serum Levels in EOC Patients

**Figure 1:**
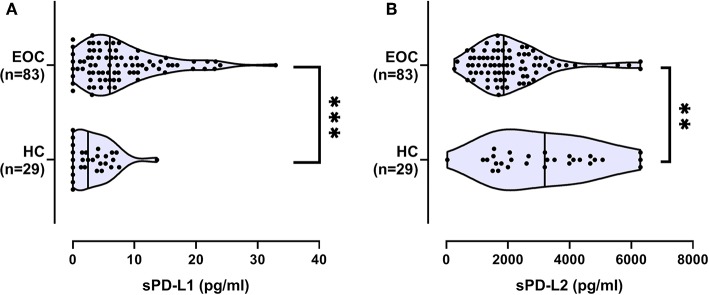
Serum levels of sPD-L1 **(A)** and sPD-L2 **(B)** in healthy controls (HC) and ovarian cancer patients (EOC). Straight line within the violin plot indicates the median. ^**^p < 0.01, ^***^p < 0.001.

Serum levels of sPD-L1 and sPD-L2 are given as median (range) pg/ml. sPD-L1 levels were significantly (*p* = 0.0001) higher in 83 EOC patients [6.0 (0–32.9)] when compared to 29 healthy females [2.5 (0–13.7); Figure 1A]. At variance to sPD-L1, the sPD-L2 serum levels of EOC patients were significantly lower 1,862 (260–6,300) (*p* = 0.003) than levels observed in healthy controls [3,193 (34–6,300); Figure 1B]. No correlation was observed between sPD-L1 levels or sPD-L2 and age in EOC patients or controls (Supplementary Figures 1A,B).

### Association of sPD-L1 and sPD-L2 Serum Levels With Clinical Characteristics

**Figure 2:**
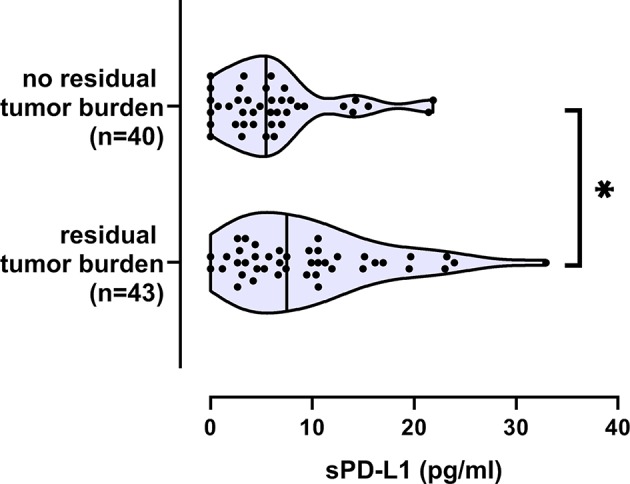
Increased sPD-L1 serum levels in EOC patients with residual tumor burden. Straight line within the violin plot indicates the median. ^*^p < 0.05.

**Figure 3:**
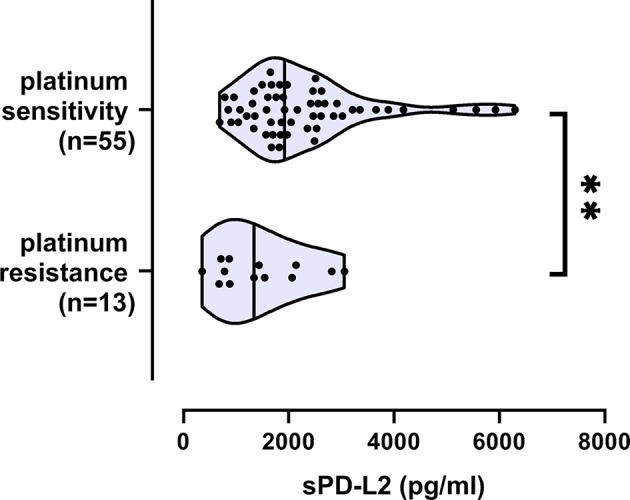
Decreased sPD-L2 serum levels in EOC patients with platinum resistance. Straight line within the violin plot indicates the median. Platinum resistance/sensitivity was available for 68 EOC patients. ^**^p < 0.01.

Concerning clinical characteristics, sPD-L1 levels and sPD-L2 did not show any association to FIGO-stage, tumor grade, lymph node infiltration, or presence of metastases (Table 1). However, increased sPD-L1 levels were significantly associated with residual tumor burden (*p* = 0.022; Table 1; Figure 2) and reduced sPD-L2 levels were significantly (*p* = 0.0096) associated with platinum-resistance (Table 1; Figure 3).

### Association of Decreased sPD-L2 Serum Levels With the Presence of CTCs

**Figure 4:**
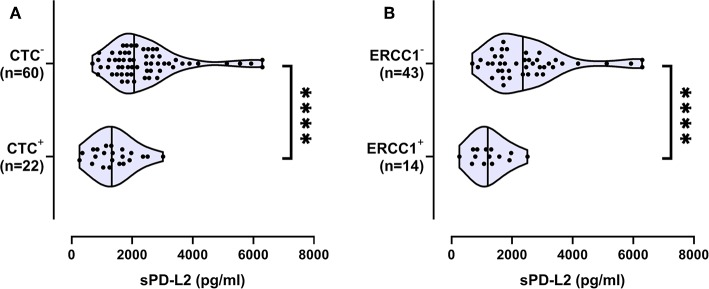
Association of decreased sPD-L2 serum levels (pg/ml) with the presence of circulating tumor cells (CTC) and the ERCC1+CTC subpopulation. Data about the presence of CTC **(A)** or ERCC1+CTC **(B)** was available for 82 and for 57 EOC patients, respectively. Straight line within the violin plot indicates the median. ^****^p < 0.0001.

The presence of CTCs before therapy was associated with lower sPD-L2 levels [1,324 (260–3,019), *N* = 22], whereas the absence of CTCs was accompanied by increased levels of sPD-L2 [2,100 (686–6,300); *p* < 0.0001; Figure 4A]. With regard to CTC subtypes, ERCC1+ CTCs were significantly associated with lower levels of sPD-L2 (*p* < 0.0001; Figure 4B). No association between the presence of CTCs and sPD-L1 was observed.

### Association of High sPD-L1 Levels With Reduced Overall and Progression-Free Survival

As shown in Table 1, samples from patients who were alive at the time point of analysis displayed significantly (*p* = 0.003) lower sPD-L1 levels [4.2 (0–23.6); *N* = 43] than samples from patients who did not survive [7.3 (1.1–32.9); *N* = 40]. Similarly, patients without disease progression exhibited lower sPD-L1 levels [4.3 (0.0–19.7); *N* = 28] than patients with progression [7.0 (0.0–32.9); *N* = 55; *p* = 0.03]. With regard to the predictive value for a 5 year PFS and OS, an optimal cut-off value of 6.4 pg/ml was calculated with a sensitivity of 58.0 or 69.25% and specificity of 76.0 or 70.4% by ROC analysis. Using this cut-off, a similar Odds-Ratio (OR) for sPD-L1 (5.37; 95% CI: 2.14–13.42) was obtained as for the presence/absence of CTC before therapy (OR: 4.61; 95% CI: 1.63–13.01, *p* = 0.003) and platinum-resistance/sensitivity (OR: 5.48; 95% CI: 1.58–19.04; *p* = 0.007) with respect to 5-year OS (Table 2A). Nevertheless, for 5 years PFS, the OR of sPD-L1 was 4-fold lower compared to the one of Platinum-resistance/sensitivity (Table 2B). No significant association of 5-year PFS or OS with sPD-L2 was observed.

**Table 2:** Association of sPD-L1, CTCs, and platinum-resistance/sensitivity status of EOC Patients with 5-year overall survival (OS) **(A)** and progression-free survival (PFS) **(B)**.

**Parameter**		**No**	**Yes**	***p*^†^**	**OR (95% CI)**
**(A)**					
**5-year OS**					
sPD-L1	>6.4 pg/mL	12	13	0.0003	5.37 (2.14–13.42)
	<6.4 pg/mL	27	31		
CTC^b^	Positive	16	6	0.003	4.61 (1.63–13.01)
	Negative^b^	22	38		
Platinum^‡^	Resistant	4	9	0.007	5.48 (1.58–19.04)
	Sensitive	38	17		
**(B)**					
**5-year PFS**					
sPD-L1	>6.4 pg/mL	34	6	0.0004	4.49 (1.61–12.47)
	<6.4 pg/mL	24	19		
CTC^b^	Positive	19	3	0.046	3.67 (1.02–13.14)
	Negative^b^	38	22		
Platinum^‡^	Resistant	13	0	0.003	21.00 (2.76–159.94)
	Sensitive	31	24		

† *p-values were calculated by Fisher's exact test, OR, Odds ratio; CI, confidence interval*.

‡ *Platinum resistance was defined as recurrence <6 months after completion of adjuvant platinum therapy*.

b *The presence of CTC in the blood was unknown for one patient*.

**Figure 5:**
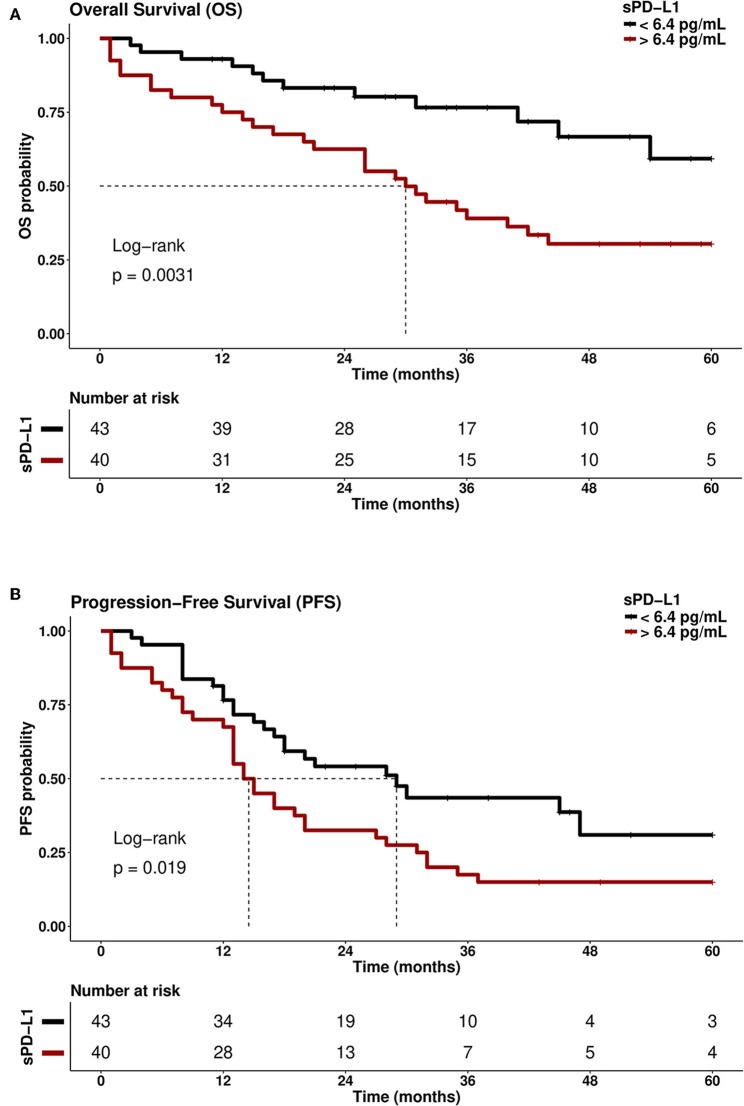
Kaplan-Meier curve of survival probability with respect to sPD-L1 serum levels (pg/ml). Patients with high sPD-L1 serum levels (>6.4 pg/mL) had a reduced **(A)** overall survival (OS; *p* = 0.0031) and **(B)** progression-free survival (PFS; *p* = 0.019) compared with patients who had low sPD-L1 levels (<6.4 pg/ml). Time was calculated from blood sampling to event (death/progression) or last follows up. Dotted line indicates the median survival time of EOC patients in the respective group.

Kaplan-Meier analysis combined with Log-rank testing revealed that patients with sPD-L1 of >6.4 pg/ml experienced a reduced OS (median OS: 30 months) compared to patients with sPD-L1 levels of <6.4 pg/ml [median OS: undefined; Hazard Ratio (HR): 2.67; 95% CI: 1.35–5.28] within an observation time of 60 months (*p* = 0.0031; Figure 5A). Considering 5-year PFS, patients with sPD-L1 <6.4 pg/ml showed a 2-fold prolonged PFS (median: 29 months) compared to patients with sPD-L1 >6.4 pg/ml (median: 14 months; *p* = 0.019, HR: 1.84; 95% CI: 1.09–3.10; Figure 5B). For CTC-negative patients, a prolonged 5-year OS (median: undefined, *p* = 0.007) and PFS (28 months; *p* = 0.053) were observed compared to CTC-positive patients (median: 26 and 15 months, respectively). As expected, patients with platinum-sensitive tumors (*N* = 55) displayed a prolonged 5-year OS (median OS: undefined, HR: 3.86; 95% CI: 1.69–8.78; *p* = 0.001) and PFS (median PFS: 32 months, HR: 20.2; 95% CI: 8.5–48.4; *p* < 0.0001) compared to patients with platinum-resistant disease (median OS: 21 months, *N* = 13; median PFS: 8 months, *N* = 13).

### High sPD-L1 Levels as a Prognostic Marker for Disease Progression and Outcome in Platinum-Sensitive EOC Patients

**Figure 6:**
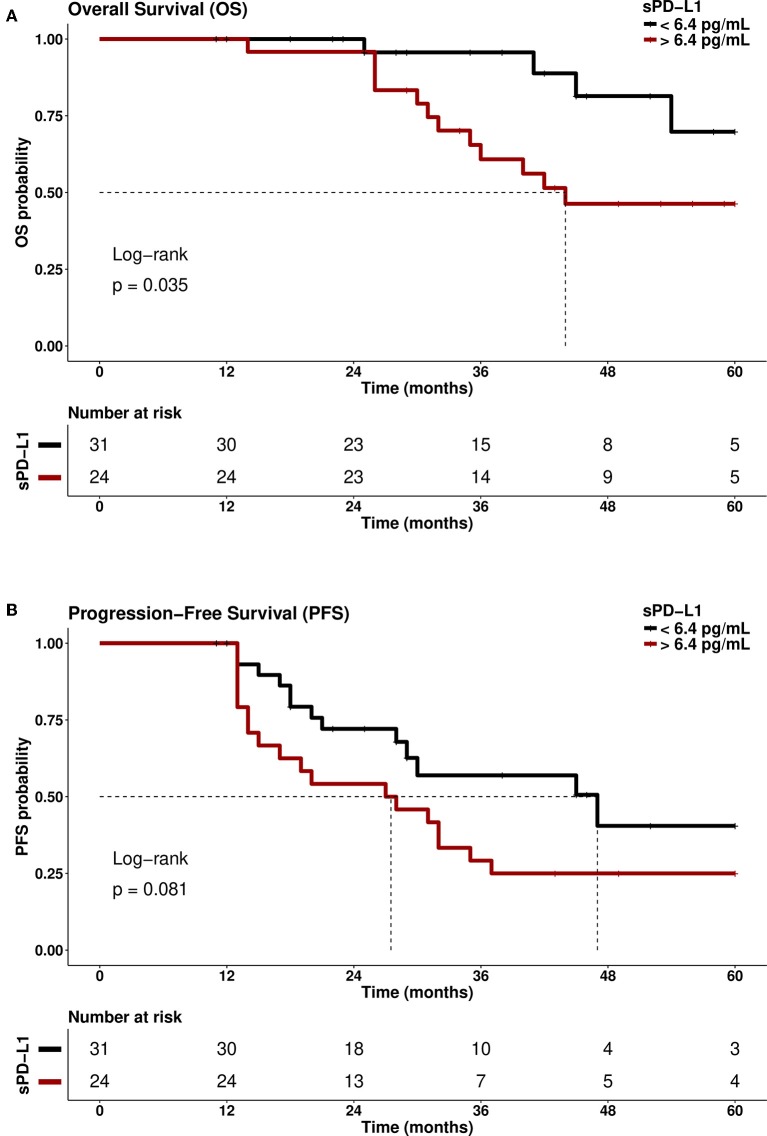
Association of high sPD-L1 levels with reduced overall survival (OS) and progression-free survival (PFS) in platinum-sensitive patients. Platinum-sensitive EOC patients with high sPD-L1 serum levels (>6.4 pg/mL) had a reduced **(A)** overall survival (OS; *p* = 0.035) and **(B)** progression-free survival (PFS; *p* = 0.081) compared with platinum-sensitive patients with low sPD-L1 levels (<6.4 pg/ml). Time was calculated from blood sampling to event (death/progression) or last follows up. Dotted line indicates the median survival time of EOC patients.

As platinum-resistance is indicative for early disease progress and reduced OS, a stratification of patients with sPD-L1 levels <6.4 and >6.4 pg/ml was performed in platinum-sensitive and in platinum-resistant patients, respectively. In the group of platinum-sensitive patients, patients with sPD-L1 levels >6.4 pg/ml showed a significantly (*p* = 0.035) reduced probability of 5-year OS (HZ: 3.96; 95% CI: 1.27–12.30) compared to patients below this cut-off (Figure 6A). A similar trend was observed for 5-year PFS (*p* = 0.083; Figure 6B): Platinum-sensitive patients having sPD-L1 levels >6.4 pg/ml presented a shortened PFS (median: 27 months) compared to patients with levels <6.4 pg/ml (median: 47 months HZ: 1.85; 95%CI: 0.91–3.79). Contrary to these findings, the 5-year OS and PFS were very similar for patients >6.4 and <6.4 pg/ml in the group of platinum-resistant patients (data not shown). Furthermore, the CTC status did not identify patients with high risk of early progression and reduced OS in the group of platinum-sensitive patients (data not shown).

## Discussion

In recent years, many inhibitory molecules and cells have been identified, which facilitate the escape of tumor cells from immune surveillance by creating an immunosuppressive microenvironment either locally at the site of the tumor or systemically. Among the different immune escape mechanisms, the interaction of PD-1 expressed on immune cells with its ligands PD-L1 or PD-L2, expressed on tumor cells or antigen presenting cells such as macrophages and dendritic cells, represents an important immune evasion pathway in EOC. In our study we focused on the diagnostic and prognostic potential of PD-L1 and PD-L2 serum levels in a cohort of 83 primary EOC patients. We were able to demonstrate different clinicopathologic significances for sPD-L1 and sPD-L2 levels: (i) sPD-L1 level was increased and sPD-L2 was decreased in EOC patients compared to healthy controls. (ii) High sPD-L1 levels were related to residual tumor burden, reduced PFS and OS, whereas low levels of sPD-L2 were associated with platinum-resistance and the presence of CTCs, especially ERCC1+ CTCs. (iii) In platinum-sensitive patients, sPD-L1 levels above 6.4 pg/ml were significantly associated with a reduced probability of 5 years OS and mildly related to a reduced 5 years PFS, while OS and PFS in platinum-resistant patients did not differ when patients were stratified according to this cut-off.

In line with our results, a recent study demonstrated enhanced sPD-L1 levels in 174 EOC patients compared to healthy women and patients with benign ovarian tumors (14). Likewise, a consistent negative effect of high sPD-L1 levels on OS has also been described in a recent meta-analysis summarizing 1,040 patients with different solid tumors including lung, gastrointestinal and renal cancer (17). While cell surface-expressed PD-L1 on macrophages has been shown to be associated with favorable prognosis in ovarian cancer (11), soluble forms of PD-L1 are thus likely to play a significant role for immune escape-mechanisms in different tumor entities. This hypothesis is further supported by a recent study demonstrating that the secretion of sPD-L1 as extracellular vesicle correlates with tumor size and inhibits the proliferation, cytokine production, and cytotoxicity of CD8 T cells in malignant melanoma (18).

Little is known about the functional consequence of cell surfaced-expressed PD-L2 or sPD-L2 in oncologic diseases. A recent meta-analysis on the correlation between PD-L2 expression and clinical outcomes in solid tumors supports the notion that high PD-L2 expression favors tumor metastasis and unfavorable prognosis in solid cancer patients after surgery, especially in hepatocellular carcinoma (19). To the best of our knowledge, PD-L2 expression at tumor sites in EOC patients has not been investigated. Only one study was able to analyze the PD-L2 expression on HLA-DR-positive cells found in ascitic fluids from EOC patients but no relationship with the clinical outcome was observed (20). In our study, EOC patients showed significantly lower sPD-L2 compared to healthy controls and within patients, those with platinum-resistant tumors showed substantially lower sPD-L2 levels than those with platinum-sensitive disease. These results suggest an important role of sPD-L2 in the host immune response to the tumor and further point to the significance of immune processes for the response to platinum therapy and outcome in EOC. Notably, low sPD-L2 levels were significantly related to the presence of ERCC1+CTCs, a CTC-subgroup associated with platinum resistance and worse outcome in EOC (16, 21). Hypothesizing a causal relationship between low sPD-L2 levels and the presence of ERCC1+CTC, one might argue that sPD-L2 mediated anti-tumor activity might play an important role in the prevention of tumor spread to the bone marrow and the elimination of CTCs.

In a previous study, we showed that immunotherapy, applying the intraperitoneal trifunctional bispecific antibody Catumaxomab, was successful in the elimination of CTCs as well as DTCs in the BM in patients with advanced ovarian cancer (7), hinting at the inverse relationship between anti-tumor immunoactivity and tumor cell spread to the BM. Other immunotherapeutic approaches have been reported to show activity in EOC such as the cytotoxic T-lymphocyte associated protein 4 (CTLA-4) antibody Ipilimumab (22). Regarding the PD-1/PD-L1/2 axis, the PD-1 antibody Nivolumab (23) as well as the anti PD-L1 antibody Atezolizumab (9) have been successfully used in EOC patients. However, little is known about the effect of these therapies on the presence of CTCs. It will be interesting to determine if the activation of different anti-tumor immune-pathways can help to eliminate CTCs as shown before for Catumaxomab.

Forthcoming, research will have to elucidate the complex mechanisms involving the cellular-expressed and soluble forms of PD-L1 and PD-L2 in EOC. However, in our study sPD-L1 helped to differentiate EOC patients from healthy controls and predict prognosis. Although our study has several limitations, mostly attributed to the small sample size and its retrospective study design, the clinical relevance of our findings is especially of interest for patients with a rather good prognosis, as defined by sensitivity to platinum therapy. In these patient cohort sPD-L1 levels helped to identify high risk patients for unfavorable disease outcome despite platinum-sensitivity, which may argue for anti-PD-1 and anti-PD-L1 antibodies treatment as additional therapeutic approaches being already successfully used in early stage clinical studies (9). Nevertheless, the value of sPD-L1 as a biomarker for the administration of anti-PD-L1/PD-1 therapy needs to be evaluated. As sPD-L2 is related with platinum sensitivity and the occurrence of CTC, both markers are promising candidates in the context of the development of new, reliable prognostic biomarkers easily accessible via liquid biopsy.

## Data Availability Statement

The datasets generated for this study are available on request to the corresponding author.

## Ethics Statement

The studies involving human participants were reviewed and approved by Ethics Committee of the University Hospital Essen. The patients/participants provided their written informed consent to participate in this study.

## Author Contributions

PB: study design, data acquisition, providing of blood and tissue samples, statistical analysis, and manuscript writing. ES: study design, serum analyses, statistical analysis, and manuscript editing. CJ: serum analyses, statistical analysis, and manuscript editing. PH: manuscript editing. RK: providing of blood and tissue samples and manuscript editing. SK-B: study design, data acquisition, providing of blood and tissue samples, and manuscript editing. VR: study design, data acquisition, serum analyses, statistical analysis, and manuscript writing.

### Conflict of Interest

SK-B was a consultant for Qiagen. VR was a consultant for Bristol-Myers Squibb. The remaining authors declare that the research was conducted in the absence of any commercial or financial relationships that could be construed as a potential conflict of interest.

## Supplementary Material

Supplementary Figure 1 No correlation between age and sPD-L1 **(A)** and sPD-L2 **(B)** serum levels in healthy controls (HC) and patients with epithelial ovarian cancer (EOC). A filled triangle indicates correlation points of HC and open circle indicate correlation points EOC patients.
