# Supplementary figures and images for: Soluble Programmed Death Receptor Ligands sPD-L1 and sPD-L2 as Liquid Biopsy Markers for Prognosis and Platinum Response in Epithelial Ovarian Cancer
Source: Front Oncol. 2019 Oct 15;9:1015. doi: 10.3389/fonc.2019.01015 (PMC6803523; doi:10.3389/fonc.2019.01015)

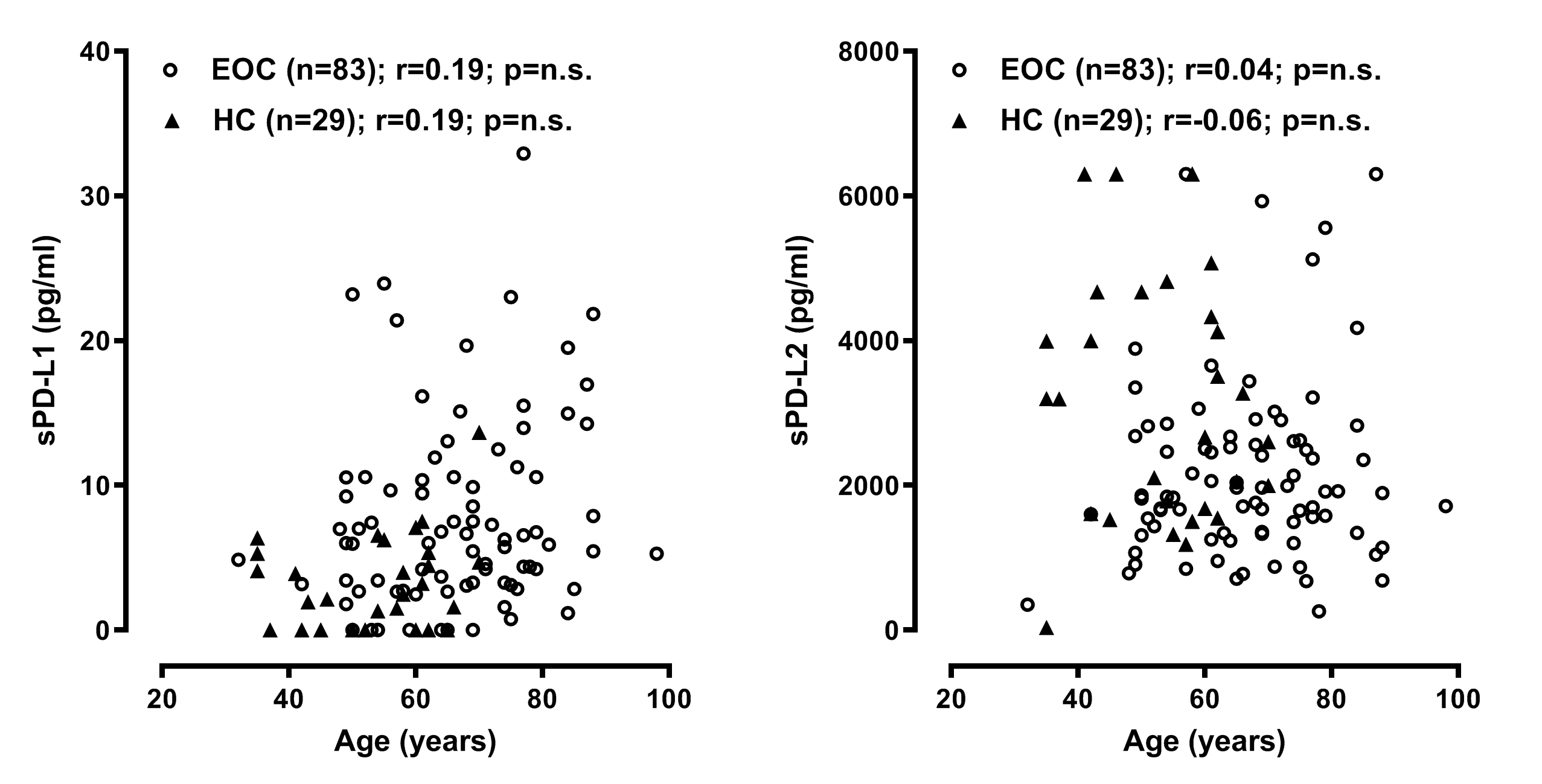

Supplement: Supplementary Figure 1 — No correlation between age and sPD-L1 (A) and sPD-L2 (B) serum levels in healthy controls (HC) and patients with epithelial ovarian cancer (EOC). A filled triangle indicates correlation points of HC and open circle indicate correlation points EOC patients. [file Image_1.TIF]
